# Knockdown of ADAM10 inhibits migration and invasion of fibroblast-like synoviocytes in rheumatoid arthritis

**DOI:** 10.3892/mmr.2022.12584

**Published:** 2022-01-04

**Authors:** Dan Li, Zhitao Xiao, Gang Wang, Xianji Song

Mol Med Rep 12: 5517-5523, 2015; DOI: 10.3892/mmr.2015.4011

Following the publication of this paper, an interested reader noted that certain of the western blotting assay data shown in Fig. 4 were strikingly similar to data appearing in different form in other articles by different authors. Owing to the fact that the contentious data in the above article had already been published elsewhere, or were already under consideration for publication, prior to its submission to *Molecular Medicine Reports*, the Editor has decided that this paper should be retracted from the Journal. After having been in contact with the authors, they agreed with the decision to retract the paper. The Editor apologizes to the readership for any inconvenience caused.

